# Facile aqueous-phase synthesis of Ag–Cu–Pt–Pd quadrometallic nanoparticles

**DOI:** 10.1186/s40580-019-0208-z

**Published:** 2019-12-02

**Authors:** Zengmin Tang, Byung Chul Yeo, Sang Soo Han, Tae-Jin Lee, Suk Ho Bhang, Woo-Sik Kim, Taekyung Yu

**Affiliations:** 1 Department of Chemical Engineering, College of Engineering, Kyung Hee University, Yongin, 17104 Republic of Korea; 2 Center for Computational Science, Korea Institute of Science and Technology (KIST), Hwarangno 14-gil 5, Seongbuk-gu, Seoul, 02792 Republic of Korea; 3 School of Chemical Engineering, Sungkyunkwan University, Suwon, 16419 Republic of Korea

**Keywords:** Ag–Cu–Pt–Pd, Quadrometallic, Janus, Short reaction time, Nanoparticles

## Abstract

Ag–Cu–Pt–Pd quadrometallic nanoparticles which small Pt and Pd nanoparticles were attached on the surface of AgCu Janus nanoparticles were firstly synthesized by sequential reduction of Pt and Pd precursor in the presence of Janus AgCu bimetallic nanoparticles as seeds in an aqueous solution. Even though there was a small amount of Cu_2_O on the surface, the synthesized nanoparticles were mainly composed of four independent metallic part, not alloy parts. By theoretical calculation and growth mechanism study, we found that different reducing rate between Ag^+^ and Cu^2+^ and sequential reduction of Pt and Pd precursors would be key roles for the formation of the quadrometallic nanoparticles.

## Introduction

Multi-metallic nanoparticles including bi- and tri- metals have been considered as a new category of attractive advanced materials due to their enhanced catalytic properties compared with their individual components [1–4]. They modified the electronic structure of metals which enables to tune the binding energy between catalysts and reaction intermediate, thus and generated the synergistic effect which can enhance the catalytic activity and durability [5–7]. These multi-metallic nanoparticles can also be a good catalyst for various tandem reactions which need more than two catalysts, and enhance the catalytic properties by formation of interconnecting area between two metals [8–10]. For example, Pd–Pt bimetallic nanoparticles has given enhanced catalytic activities for various catalytic reactions including photocatalytic water splitting, oxygen reduction, hydrogen evolution, preferential oxidation, selective heterogeneous hydrogenation, hydrogen peroxide generation, and alcohol oxidation [11–14]. Ag–Cu bimetallic nanoparticles also have attracted more attention due to their high electron conductivity, active antibacterial property, and optical property [15–17]. Unfortunately, the process of manufacturing multi-metallic nanoparticles more than three components has not been developed much in the previous studies. Therefore, in this study, we tried to develop a method for easily fabricating multi-metallic nanoparticles with more than four metallic configurations. In order to produce economically viable nanoparticles for catalysts, we designed Ag–Cu–Pt–Pd quadrometallic nanoparticles with inexpensive Ag and Cu inside and expensive Pt and Pd outside.

In the past few decades, there have been lots of solution-based synthetic processes for multi-metallic nanoparticles including thermal decomposition, co-reduction, galvanic replacement, and seed-mediated growth [11, 18–21]. To prepare multi-metallic nanoparticles, thermal decomposition and co-reduction method typically use to separate the reaction rate between two metal precursors. For example, Ni@Pd core–shell nanoparticles were synthesized by slowly heating an organic solution containing Ni and Pd precursors [22]. Because of different decomposing temperature, Ni formed a core and Pd was located in shell. The galvanic replacement reaction which uses reduction potential difference between two metals is typically used to make noble bimetallic nanoparticles including Ag–Au, Cu–Pd, and Pd–Pt [23–25]. However, there is an economical problem to use it for a large-scale catalyst production process because we have to use one metal as a sacrificial template to make bimetallic nanoparticles. To prepare Ag–Cu–Pt–Pd quadrometallic nanoparticles in this study, we introduced both co-reduction and seed-mediated growth. Compared with thermal decomposition, co-reduction method has a mild reaction condition such as low reaction temperature, air atmosphere, and do not use of toxic organic solvent [26]. First, we synthesized Janus AgCu bimetallic nanoparticles using co-reduction method. Because the Janus AgCu nanoparticle has two independent metals, not alloy form, we could expect to form Pt and Pd nanoparticles on the different places of the AgCu nanoparticles due to the different standard reduction potential. Therefore, Janus AgCu nanoparticle was used as mother nanoparticles, then attaching Pt and Pd nanoparticles on the surface of the Janus AgCu bimetallic nanoparticles using seed-mediated growth, thus forming the Ag–Cu–Pt–Pd quadrometallic nanoparticles. All of the synthetic processes were performed in an aqueous-phase, and reaction time is really short, only 10 min. In addition, we studied the growth mechanism of the quadrometallic nanoparticles by using theoretical calculation and monitoring the growth behavior.

## Experimental methods

### Materials

Silver nitrate (AgNO_3_), cupric sulfate pentahydrate (CuSO_4_·5H_2_O), potassium tetrachloroplatinate (K_2_PtCl_4_, 99.99%), sodium tetrachloropalladate (Na_2_PdCl_4_, 98%), branched polyethyleneimine (BPEI, MW = 750,000, 50 wt % solution in water), L-ascorbic acid (C_6_H_8_O_6_, ≥ 99%), and polyvinyl pyrrolidone (PVP, MW = 10,000) were purchased from Sigma-Aldrich. All chemicals were used as received without further purification.

### Synthesis of Janus AgCu bimetallic nanoparticles

0.2 mL of BPEI aqueous solution (0.2 g/mL), 0.1 mL of CuSO_4_ aqueous solution (1 M), and 0.02 mL of AgNO_3_ aqueous solution (1 M) were dissolved in 3 ml of water and heated to 80 °C. 3 mL of L-ascorbic acid aqueous solution (0.2 g/mL) was then added and the resulting solution was heated at same temperature for 5 min. The final product was collected by centrifugation and washed by water with several times.

### Synthesis of Ag–Cu–Pt–Pd metallic nanoparticle

After washing process, obtained Ag–Cu nanoparticles were dispersed in a 5 mL of aqueous solution containing l-ascorbic acid (5 mg) and PVP (2.5 mg) and stirred at room temperature. 100 µL of K_2_PtCl_4_ (0.01 M) solution was added into the solution using a pipette. After 5 min, 100 µL of Na_2_PdCl_4_ (0.01 M) solution was then injected. After reacting 5 min more, and final product was separated by centrifugation and wash one time by water.

### Characterization

Transmission electron microscopy (TEM) and energy-dispersive X-ray spectroscopy (EDS) were carried out by using a JEOL JEM-2100F transmission electron microscope at 200 kV. X-ray diffraction (XRD) patterns were measured by using a Rigaku D-MAX/A diffractometer at 35 kV and 35 mA. UV–vis measurements were conducted on a Shimadzu 2550 spectrophotometer. Inductively Coupled Plasma Spectrometer (ICP) analyses were performed by using a Direct Reading Echelle ICP, LEEMAN.

### Density functional theory calculation

To understand thermodynamics of the AgCu bimetallic system, we performed density functional theory (DFT) calculations. All the calculations were performed using the Vienna Ab Initio Simulation Package (VASP) [27] using the projector-augmented-wave (PAW) method [28] to describe the potential from the ionic core. For the exchange and correlation terms, we employed the revised Perdew-Burke-Ernzerhof (RPBE) functional [29]. An energy cutoff of 400 eV and Monkhorst–Pack k-point meshes of 8 × 8 × 8 for the bulk calculations were used after an extensive convergence test. All of the crystal structures were optimized until the energy change was less than 1 × 10^−6^ eV per cell and the force on each atom was less than 0.01 eV/Å.

## Results and discussion

**Scheme 1:**
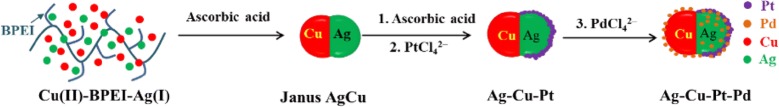
Schematic illustration of synthetic procedure of Cu–Ag–Pt–Pd nanoparticle

**Fig. 1:**
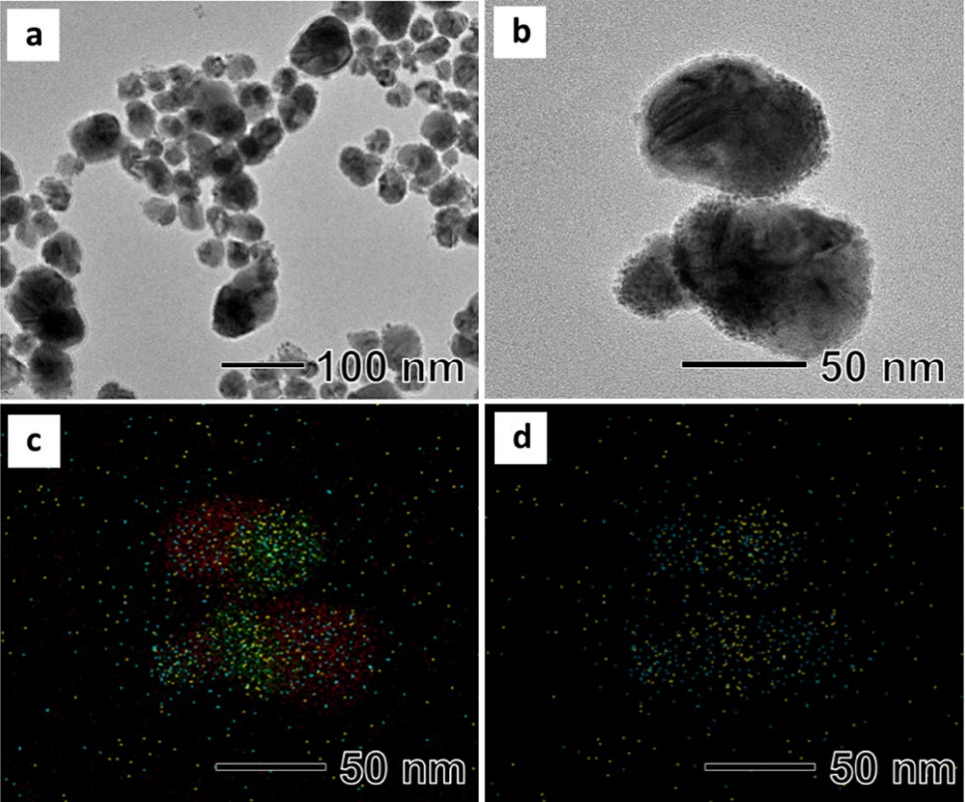
**a**, **b** TEM images of synthesized Ag–Cu-Pt–Pd nanoparticle, **c**, **d** EDS mapping images of metallic elements distribution in synthesized Ag–Cu–Pt–Pd nanoparticles (red refers to Cu, green refers to Ag, yellow refers to Pt, and blue refers to Pd)

Ag–Cu–Pt–Pd quadrometallic nanoparticles were synthesized by the sequential reduction of Pt and Pd precursor in the presence of AgCu bimetallic nanoparticles as seeds (Scheme 1). Color of the AgCu bimetallic nanoparticles suspension was red and quickly changed to dark red and dark after addition of Pt and Pd precursor, respectively. The total reaction time was really short, only 10 min. Figure 1a shows a typical TEM image of the synthesized Ag–Cu–Pt–Pd quadrometallic nanoparticles, revealing the formation of elliptical nanoparticles with an average long axis length of around 89.1 nm (Additional file 1: Fig. S1a). The nanoparticles consisted of two major parts, AgCu bimetallic nanoparticles and small Pd and Pt nanoparticles attached on the surface of the AgCu nanoparticles (Fig. 1b). The AgCu nanoparticles had a Janus morphology with 40 nm-sized Ag and Cu attached to each other. And small Pd and Pt nanoparticles with sizes of around 2 nm were well dispersed on the surface of the AgCu nanoparticles. The EDS mapping image of the Ag–Cu–Pt–Pd quadrometallic nanoparticles clearly show that small Pd and Pt nanoparticles were attached the surface of the Janus AgCu nanoparticles (Fig. 1c). Interestingly, we found that Pt nanoparticles were only attached on the Ag part, while Pd nanoparticles were found on all surface of the AgCu nanoparticles (Fig. 1d). The XRD result (Additional file 1: Fig. S1b) shows that the nanoparticles were mainly composed of face-centered cubic (*fcc*) Cu (Fm3 m, a = 3.615 Å, Joint Committee on Power Diffraction Standard (JCPDS) #04-0836) and *fcc* Ag (Fm3 m, a = 4.086 Å, JCPDS #04-0783). We did not find diffraction peaks of Pt and Pd, possibly due to the low atomic ratio. The amount of Pd and Pt in the nanoparticles characterized by an ICP analysis were 0.68 wt% for Pd and 1.26 wt% for Pt, respectively (Additional file 1: Table S1). In addition, weak diffraction peaks were observed at 36.5°, 42.5°, and 61.7°, respectively, indicating that there was small amount of Cu_2_O in the nanoparticles (Pn3 m, a = 4.269 Å, JCPDC #05-0667). We think that Cu in the surface of the nanoparticles would be oxidized by oxygen in air.

**Fig. 2:**
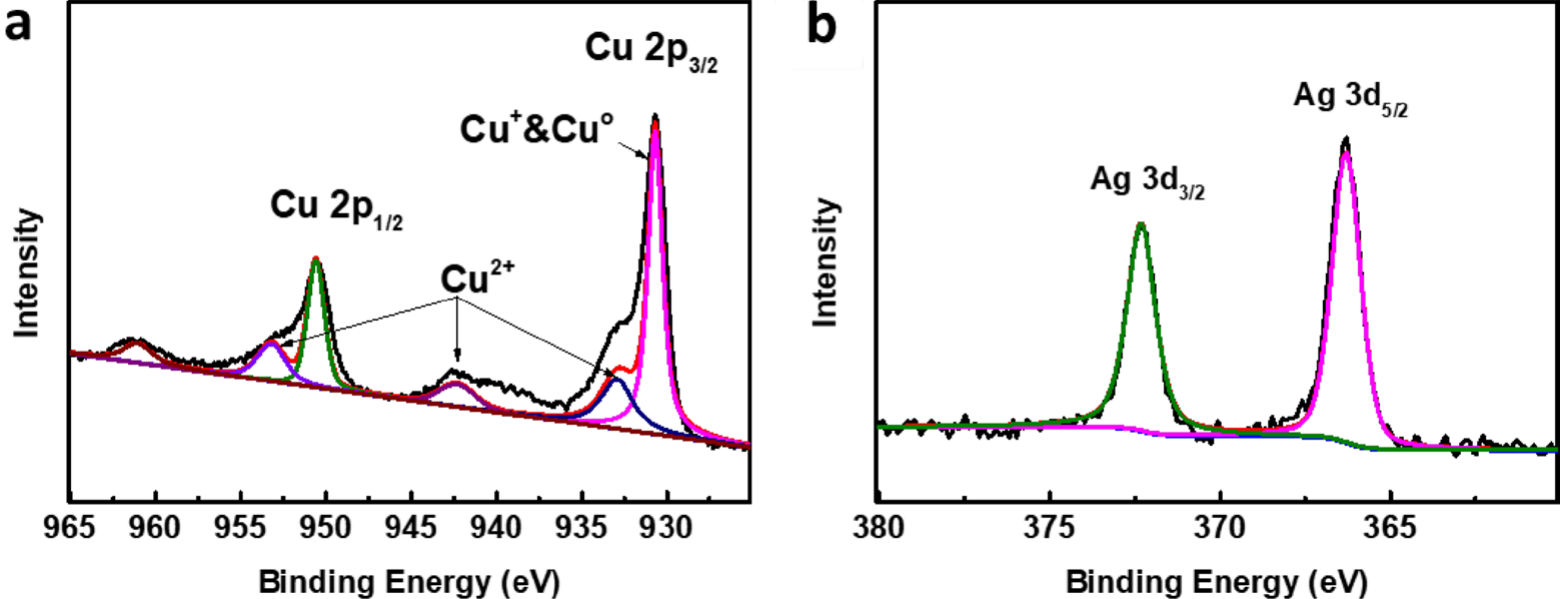
XPS spectrum of Cu-Ag-Pt–Pd nanoparticles (**a**) Cu and (**b**) Ag

To further confirm the oxidation states of Cu in the nanoparticles, XPS analyses were performed (Fig. 2). The Cu XPS 2p core level spectrum had two sets of 2p peaks, one set had Cu 2p_3/2_ and Cu 2p_1/2_ peaks at 930.7 and 950.6 eV, respectively, corresponding to existence of copper with low valance, including Cu and Cu^+^ (Fig. 2a) [30]. Meanwhile, the weak peaks of Cu 2p_3/2_ at 932.66 eV and Cu 2p_1/2_ at 952.89 eV in combination with the satellite peaks at 942.16 eV are typical characteristics of Cu^2+^, which originate from the inevitable oxidation of Cu/Cu^+^ species in these nanoparticles. Compared with Cu, the Ag 3d core level spectrum exhibits only a couple of peaks at 366.3 eV for Ag 3d_5/2_ and 372.3 eV for Ag 3d_3/2_, respectively, showing the presence of only metallic Ag in the nanoparticles (Fig. 2b). These results are well supported the XRD result, which demonstrate that a small amount of Cu_2_O is present on the surface of the nanoparticles.

**Fig. 3:**
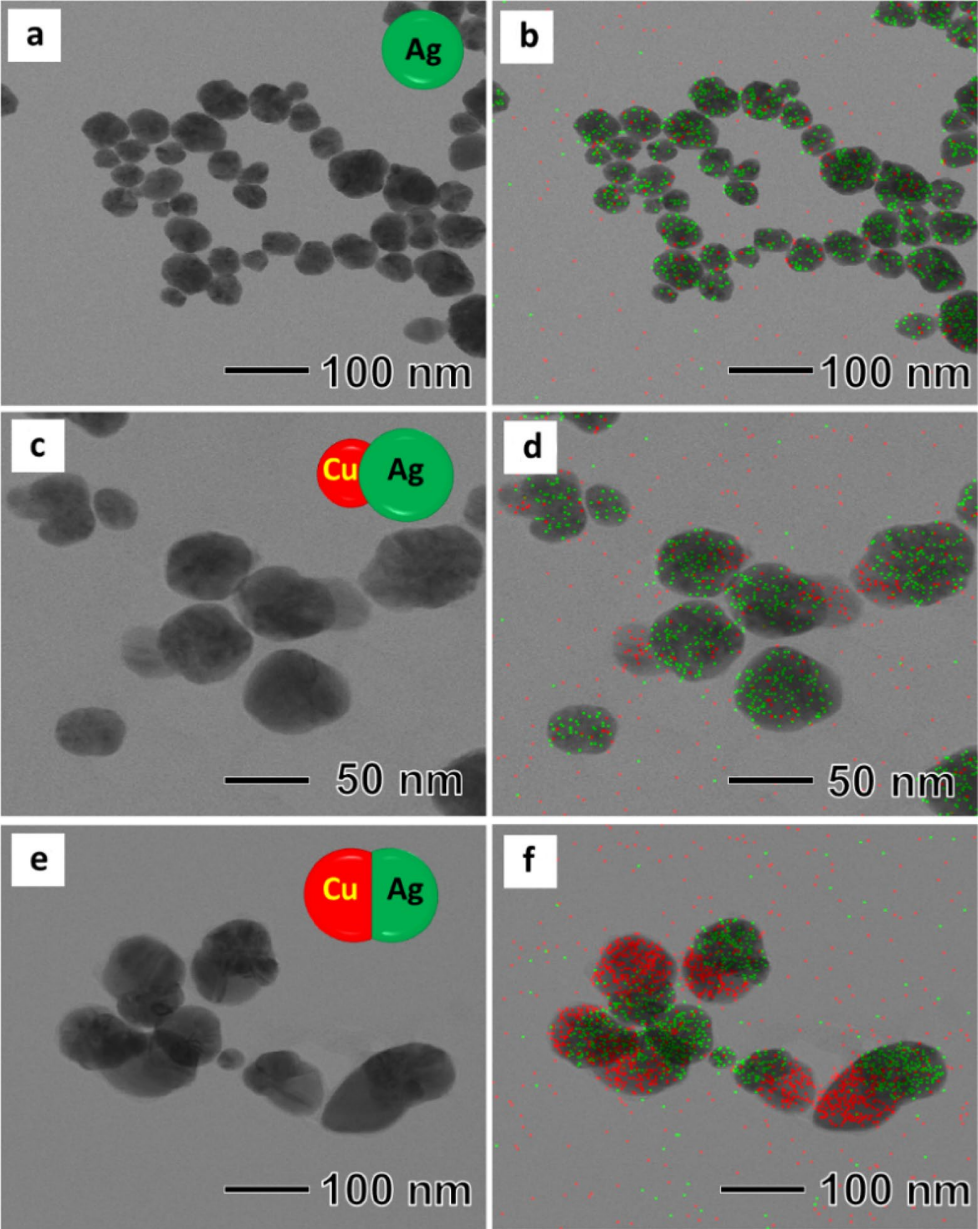
TEM images and EDS images of products obtained at different reaction time (**a**) and (**b**) 5 s, (**c**) and (**d**) 30 s, (**e**) and (**f**) 150 s

The present synthesis was composed of two steps, synthesis of Janus AgCu bimetallic nanoparticles and attaching Pd and Pt nanoparticles on the surface of the AgCu nanoparticles. The Janus AgCu bimetallic nanoparticles were synthesized by co-reducing Ag and Cu precursor using L-ascorbic acid in the presence of BPEI as a stabilizer in an aqueous solution. For better understanding of the formation of the Janus nanoparticles instead of AgCu alloy, an aliquot of the reaction solution was taken out at the early stages and observed by using TEM and EDS. 5 s after the injection of L-ascorbic acid, spherical nanoparticles with size of around 40 nm were found as shown in Fig. 3a. EDS mapping analysis revealed that the synthesized nanoparticles were Ag, not Cu (Fig. 3b). As the reaction proceeded to t = 30 s, we observed the formation of single Cu bump with sizes of around 10 nm on each Ag nanoparticle, as shown in Fig. 3c, d. During the next 150 s, the size of the Cu bump further increased to around 40 nm, thus forming peanut-shaped Janus nanoparticles (Fig. 3e, f). UV spectrum of an aqueous suspension of the synthesized nanoparticles shows the presence of two peaks at 409 nm and 577 nm, which correspond to Ag and Cu nanoparticles, respectively, demonstrating the synthesized nanoparticles has AgCu Janus structure, not alloy form (Additional file 1: Fig. S2). We think that the formation of Janus nanoparticles is caused by two reasons, difference of the reducing rate between Ag^+^ and Cu^2+^, and instability of AgCu alloy. Due to the high redox potential of Ag^+^ to Ag (0.799 V) compared with Cu^2+^ to Cu (0.34 V), the Cu nanoparticles could not be formed before reducing all of Ag^+^ in the solution [26]. After the formation of Ag nanoparticles, Cu nanoparticles started to grow the surface of the Ag nanoparticles by heterogeneous nucleation, thus forming the Janus-shaped nanoparticles.

To support the Janus-shaped structure formation of the Ag–Cu nanoparticles, we performed DFT calculations. For the composition of Ag:Cu = 1:1, ten thermodynamic phases (L1_0_, B2, B1, B4, B3, B33, B11, B19, B27, and L1_1_) were investigated, and then their formation energies (E_f_) relative to pure Ag and Cu crystals were calculated (Table 1). All of the ten phases have positive E_f_, indicating that the binary system is thermodynamically immiscible, i.e., separated domains of each element are preferably dominate rather than homogenously mixing or alloying of two elements at the atomic level. Indeed, according to a phase diagram of Ag–Cu binary system [31], the two elements are completely immiscible in the full composition ranges at the synthesis temperature (80 °C) of the Janus-shape Ag–Cu nanoparticles.

**Table 1:** Calculated formation energy of each candidate for alloyed Cu–Ag system with a composition of 50:50

Crystal structures		E_f_ (eV)
L1_0_		0.22
B2		0.23
B1		0.81
B4		1.94
B3		3.27
B33		3.71
B19		5.43
B11		6.13
B27		3.04
L1_1_		3.48

Cu atom: orange color, Ag atom: grey color

**Fig. 4:**
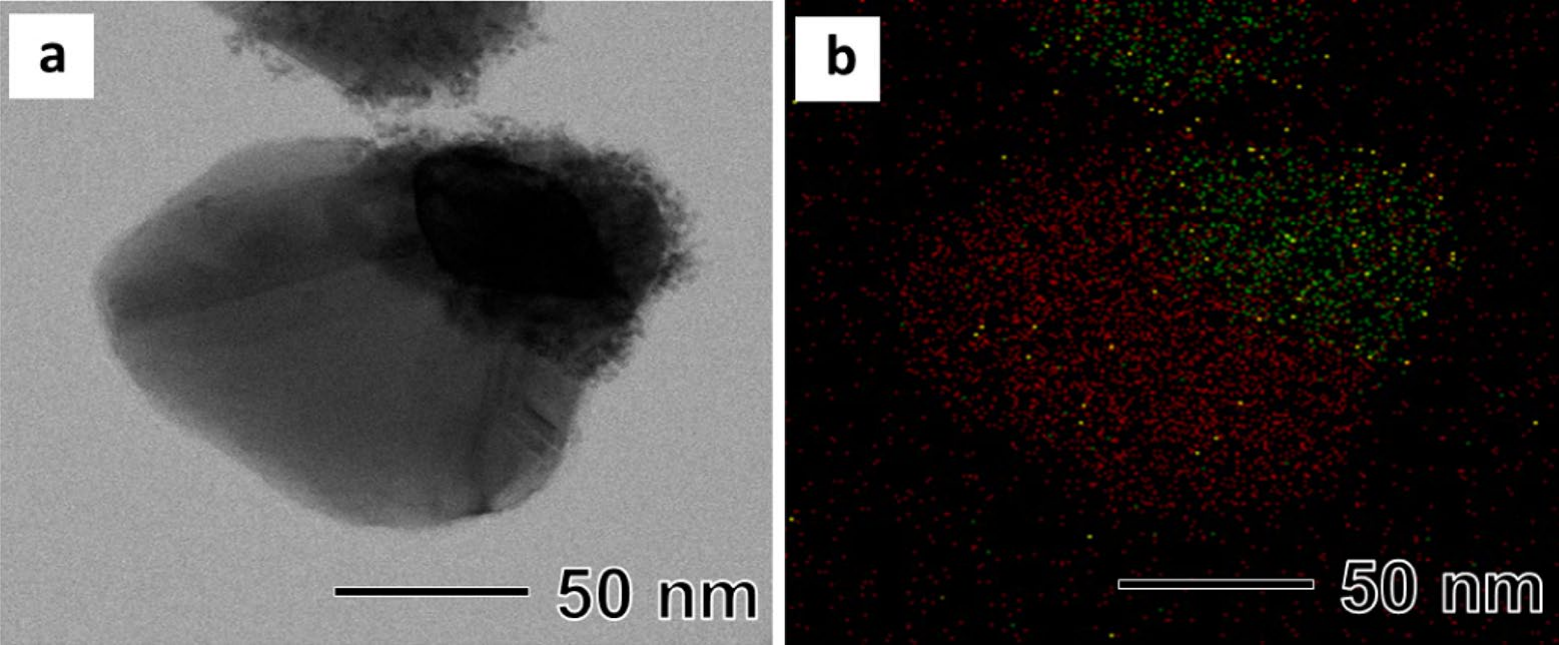
**a** TEM image and **b** EDS-mapping image of metallic elements distribution in Ag–Cu–Pt nanoparticles

To attach Pt and Pd nanoparticles on the surface of the Janus AgCu nanoparticles, we tried to attach Pt first rather than Pd in order to avoid the galvanic replacement reaction between Pd and PtCl_4_^2−^. TEM image of the sample after reducing Pt precursor with l-ascorbic acid in the presence of the AgCu nanoparticles shows that small nanoparticles with sizes of around 2 nm were dispersed on the surface of only Ag part of the AgCu nanoparticles (Fig. 4). We postulate that the chemical potential difference between Ag and Cu causes electron accumulation on the surface of the Ag part, therefore Pt would be reduced preferentially on the Ag surface which had a relatively high electron density distribution [14]. However, Pd nanoparticles were attached on all surface of the AgCu nanoparticles, unlike Pt. A complete understanding of the attachment of Pd and Pt nanoparticles on the surface of AgCu nanoparticles may require further studies through not only experiments but also simulations.

## Conclusions

We have first demonstrated that an aqueous-phase synthetic route to Ag–Cu–Pt–Pd quadrometallic nanoparticles which small Pt and Pd nanoparticles were attached on the surface of AgCu Janus nanoparticles. The meaning of this study can be summarized in two parts. We have produced quadrometallic nanoparticles having four independent metal domains in one particle without any alloy parts using different reducing rate and sequential reduction of each precursor. In addition, the reaction time for these complex nanoparticles was really short, 5 min for Janus AgCu nanoparticles and more 5 min for the Ag–Cu–Pt–Pd quadrometallic nanoparticles. Therefore, we expect that our newly developed synthetic process can be used for various multi-metallic nanoparticle manufacturing processes.

## Supplementary information

**Additional file 1: Figure S1.** (a) size distribution of nanoparticles, (b) XRD patterns of synthesized Cu-Ag-Pt-Pd nanoparticles. **Figure S2.** UV-vis spectrum of prepared Ag-Cu bimetallic nanoparticles. **Table S1.** ICP data of Cu-Ag-Pt-Pd nanoparticles.

## Data Availability

Not applicable.
