# Supplementary material for: Facile aqueous-phase synthesis of Ag–Cu–Pt–Pd quadrometallic nanoparticles
Source: Nano Converg. 2019 Dec 2;6:38. doi: 10.1186/s40580-019-0208-z (PMC6885459; doi:10.1186/s40580-019-0208-z)
Supplement: Supplementary file 1 — Additional file 1: Figure S1. (a) size distribution of nanoparticles, (b) XRD patterns of synthesized Cu-Ag-Pt-Pd nanoparticles. Figure S2. UV-vis spectrum of prepared Ag-Cu bimetallic nanoparticles. Table S1. ICP data of Cu-Ag-Pt-Pd nanoparticles. [file 40580_2019_208_MOESM1_ESM.docx]

**Additional file**

**Facile aqueous-phase synthesis of Ag-Cu-Pt-Pd quadrometallic nanoparticles**

Zengmin Tang ^1^, Byung Chul Yeo ^2^, Sang Soo Han ^2^, Tae-Jin Lee^3^, Suk Ho Bhang^3^, Woo-Sik Kim ^1,^*, Taekyung Yu ^1,^*

*^1^ Department of Chemical Engineering, College of Engineering, Kyung Hee University, Yongin 17104, Republic of Korea*

*^2^ Center for Computational Science, Korea Institute of Science and Technology (KIST), Hwarangno 14-gil 5, Seongbuk-gu, Seoul 02792, Republic of Korea*

*^3^ School of Chemical Engineering, Sungkyunkwan University, Suwon 16419, Republic of Korea*

***** Correspondence: wskim@khu.ac.kr, tkyu@khu.ac.kr.


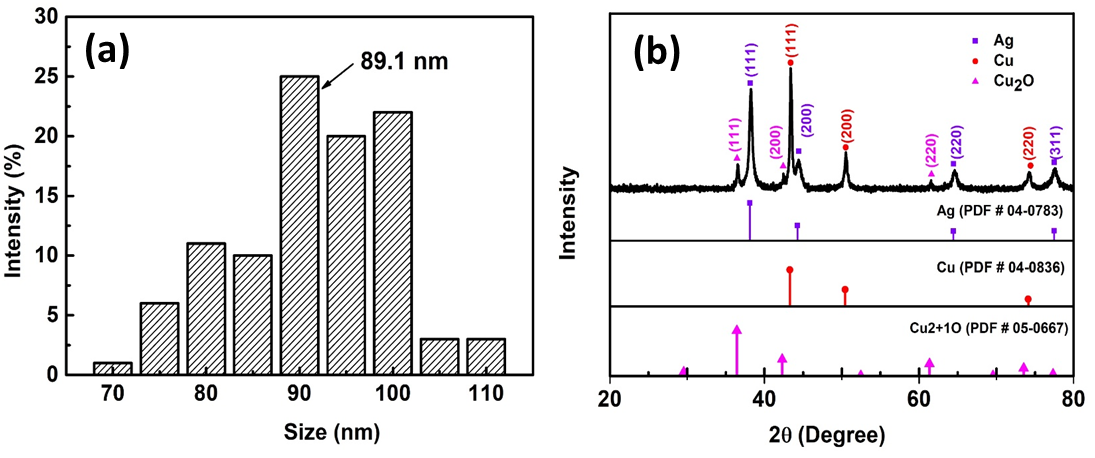


**Figure S1**. (a) size distribution of nanoparticles, (b) XRD patterns of synthesized Cu-Ag-Pt-Pd nanoparticles.

Table S1. ICP data of Cu-Ag-Pt-Pd nanoparticle

| Sample | Metal | Mass concentration | percentage |
| --- | --- | --- | --- |
| Cu-Ag-Pt-Pd | Pd | 1.92 mg/L | 0.68% |
|  | Pt | 3.55 mg/L | 1.26% |
|  | Ag | 96.9 mg/L | 34.32% |
|  | Cu | 180 mg/L | 63.74% |


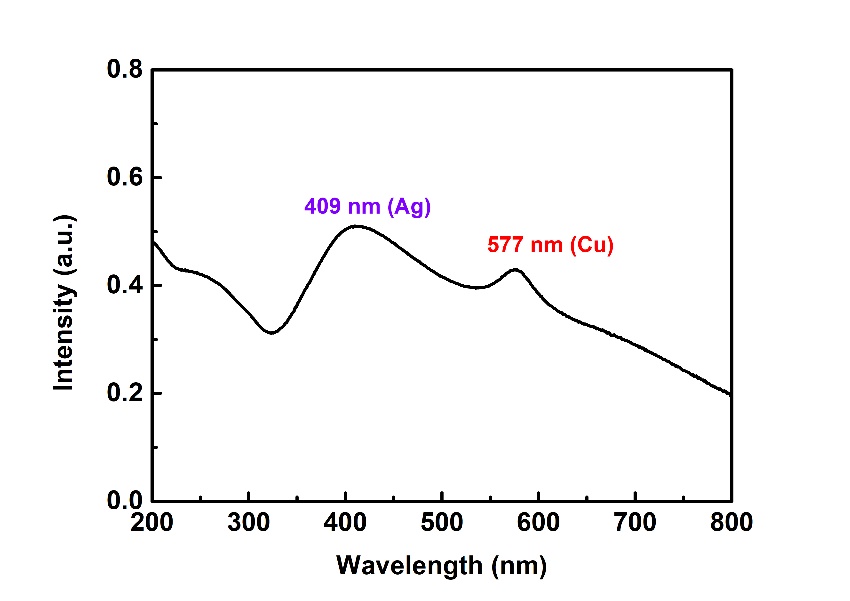


Figure S2. UV-vis spectrum of prepared Ag-Cu bimetallic nanoparticles.
